# Serum and Salivary IgA, IgG, and IgM Levels in Oral Lichen Planus: A Systematic Review and Meta-Analysis of Case-Control Studies

**DOI:** 10.3390/medicina54060099

**Published:** 2018-12-03

**Authors:** Hamid Reza Mozaffari, Elisa Zavattaro, Abas Abdolahnejad, Pia Lopez-Jornet, Neda Omidpanah, Roohollah Sharifi, Masoud Sadeghi, Mohammad Shooriabi, Mohsen Safaei

**Affiliations:** 1 Department of Oral and Maxillofacial Medicine, School of Dentistry, Kermanshah University of Medical Sciences, Kermanshah 6713954658, Iran; mozaffari20@yahoo.com (H.R.M.); n.omidpanah@yahoo.com (N.O.); 2 Medical Biology Research Center, Kermanshah University of Medical Sciences, Kermanshah 6714415185, Iran; 3 Dermatology Unit, Department of Translational Medicine, University of Eastern Piedmont “Amedeo Avogadro”, 28100 Novara, Italy; elisa.zavattaro@med.uniupo.it; 4 Students Research Committee, Kermanshah University of Medical Sciences, Kermanshah 6715847141, Iran; abbas_a@icloud.com; 5 Facultad de Medicina y Odontologia Universidad de Murcia, Hospital Morales Meseguer, Clinica Odontologic Adv Marques Velez s/n, 30008 Murcia, Spain; majornet@um.es; 6 Department of Endodontics, School of Dentistry, Kermanshah University of Medical Sciences, Kermanshah 6713954658, Iran; roholahsharifi@gmail.com; 7 Department of Oral Medicine, Faculty of Dentistry, Ahvaz Jundishapur University of Medical Sciences, Ahvaz 6135715794, Iran; dsshoriabii@yahoo.com; 8 Oral and Dental Sciences Research Laboratory, School of Dentistry, Kermanshah University of Medical Sciences, Kermanshah 6713954658, Iran; mohsen_safaei@yahoo.com

**Keywords:** oral lichen planus, immunoglobulin, serum, saliva

## Abstract

Immunoglobulins (IgA, IgG, and IgM) are significant anti-inflammatory factors. The meta-analysis aimed to assess the serum and salivary levels of Igs as more important immunoglobulins in patients affected by oral lichen planus (OLP) compared to the healthy controls. Four databases, including PubMed/Medline, Scopus, Web of Science, and Cochrane Library as well as Iranian databases were checked up to January 2018 without language restriction. The quality of each involved study was done using the Newcastle–Ottawa Quality Assessment Scale (NOS) questionnaire. A random-effects model analysis was done by RevMan 5.3 software applying the mean difference (MD) plus 95% confidence intervals (CIs). The CMA 2.0 software was applied to calculate the publication bias among the studies. Out of 70 studies found in the databases, 8 studies were involved and analyzed in the meta-analysis. The meta-analysis included 282 OLP patients and 221 healthy controls. The pooled MDs of serum levels of IgA, IgG, and IgM were −0.13 g/L [95% CI: −0.24, −0.02; *P* = 0.02], 1.01 g/L [95% CI: −0.91, 2.93; *P* = 0.30], and −0.06 g/L [95% CI: −0.25, 0.14; *P* = 0.56], respectively; whereas, the salivary IgA and IgG levels were 71.54 mg/L [95% CI: 12.01, 131.07; *P* = 0.02] and 0.59 mg/L [95% CI: −0.20, 1.38; *P* = 0.14], respectively. Considering the few studies performed on saliva, the results suggested that the salivary levels, especially IgA level had higher values than the serum levels. Therefore, the salivary immunoglobulins can play a significant function in the OLP pathogenesis.

## 1. Introduction

Oral lichen planus (OLP) is a chronic inflammatory disorder associated with various other systemic disorders [1]. The prevalence of this disorder in the general population changes from 1% to 2% [2]. The disease is more frequent in women than in men; furthermore, it can also involve the genital area [3]. In its ulcerative and sclerosus varians, lichen planus (LP) represents a precancerous lesion with potential risk of malignant transformation, mainly towards squamous cell carcinoma [4]. Local immune factors may have a function in protection against oral diseases, and these defenses may be related to responses of immunoglobulin (Ig) [5]. IgA and IgG are the most important antibodies in the serum and IgA has the highest daily synthesis rate [6]. IgA is distributed distinctly between the systemic and mucosal immune system and has a key function in protecting immunity [7]. IgG molecules are a group of glycoproteins extremely important for supporting the body against the invading pathogens [6]. IgM is the first antibody made throughout a primary antibody response, and is predominantly created by B-1 cells [8]. These Ig have a strong anti-inflammatory effect [5,6,7,8]. On this basis, the role of immunoglobulin levels has been studied as a possible biomarker of OLP, thus its possible diagnostic and/or prognostic role has been proposed, with controversial results [9,10]. Saliva as a diagnostic tool has a number of advantages in comparison with serum tests and other diagnostic tests, including being easily collected, non-invasive, accessible, safe, and precise [11]. Therefore, the objective of the meta-analysis was to evaluate the serum and salivary levels of IgA, IgG, and IgM in the OLP patients as compared to the healthy controls.

## 2. Materials and Methods

This meta-analysis was done based on the guidelines for the PRISMA [12].

## 3. Search Strategies

A comprehensive search was done in four databases, including PubMed/Medline, Scopus, Web of Science, Cochrane Library as well as Iranian databases with key terms (“oral lichen planus” or “OLP”), (“immunoglobulin”, “IgA”, “IgG”, or “IgM”), and (“saliva”, “salivary, or “serum”) up to January 2018, without language restriction.

## 4. Study Selection

One author (M.S.) evaluated the studies to investigate if they met the inclusion criteria. The second author (H.R.M.) re-checked the studies with the mentioned criteria. The inclusion criteria for selecting the studies were: (i) detection of IgA, IgG and/or IgM levels in the serum and/saliva of OLP patients in case-control studies; (ii) the OLP diagnosis was in accordance with the clinical and/or histopathological WHO criteria [10]; (iii) healthy controls were reported and no other skin and/or systemic diseases affected OLP patients.

## 5. Data Extraction

The relevant information extracted from every study was: the name of author, the year of publication, country, the number of OLP patients and healthy controls, male percent and the mean age of OLP patients and healthy controls, levels of Ig in two groups, detection method, and Ig values.

## 6. Quality Evaluation

One author (M.S.) measured the quality of each involved study applying the Newcastle–Ottawa Quality Assessment Scale (NOS) with a maximum total score of 9 for a case-control study [13]; with a score ≥7 being high quality.

## 7. Statistical Analyses

A continuous analysis (random-effects model) was done by Review Manager 5.3 software (RevMan 5.3, The Cochrane Collaboration, Oxford, UK), using mean difference (MD) plus 95% confidence intervals (CIs). The pooled MD of the studies was calculated to estimate serum or salivary Ig levels of OLP patients as compared with the healthy controls. The Q and *I*^2^ statistics were used to check heterogeneity between estimations. For the Q statistic, heterogeneity was supposed if *P* < 0.1. *P*-value (two-tailed) < 0.05 was supposed statistically significant. The CMA 2.0 software (CMA 2.0, Biostat Inc., Englewood, NJ, USA) was applied to calculate the publication bias between the studies by funnel plot, as well as Begg’s and Egger’s tests. The unit of measurement of Ig was g/L in serum and mg/L in saliva.

## 8. Results

**Figure 1:**
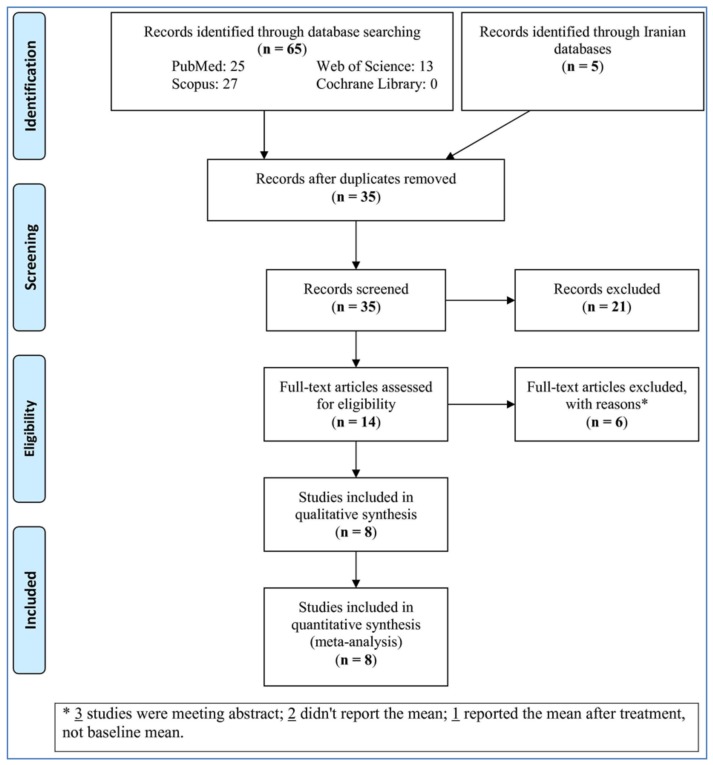
Flow-chart of the study.

Out of 70 studies found in the databases, 35 studies were screened after removing the duplicate studies, 21 of which were not relevant and were excluded. After that, the full-texts of 14 studies were evaluated for eligibility, 6 of which were omitted with the reasons that are reported in Figure 1. Finally, eight studies were involved and analyzed in the meta-analysis.

Some characteristics of the eight studies involved in the meta-analysis are reveled in Table 1. The studies were reported from 1982 to 2016; two studies from Iran [14,15], one study from Scotland [16], one from Greece [17], one from Croatia [18], one from Sweden [19], one from India [20], and one from Spain [10]. The meta-analysis included 282 OLP patients and 221 healthy controls. IgA was measured in serum and saliva in five studies [14,15,16,17,19,20] and three studies [10,14,18], respectively; whereas, IgG was measured in serum and saliva in five studies [15,16,17,19,20] and two studies [14,18], respectively. Serum IgM was measured in four studies [15,16,17,19] without reporting the saliva. The detection methods of Ig are shown in Table 1.

**Figure 2:**
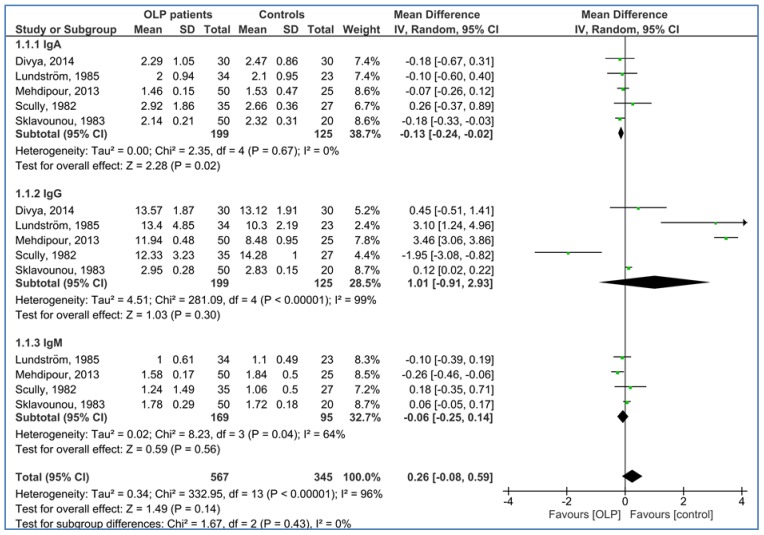
Forest plot of random-effects of serum immunoglobulins (IgA, IgG, and IgM) levels in oral lichen planus (OLP) patients compared to the healthy controls.

Figure 2 illustrates the serum levels of Igs in the OLP patients as compared to the healthy controls. The pooled MDs of serum levels of IgA, IgG, and IgM were −0.13 g/L [95% CI: −0.24, −0.02; *P* = 0.02; *I*^2^ = 0% (*P*_h_ = 0.067)], 1.01 g/L [95% CI: −0.91, 2.93; *P* = 0.30; *I*^2^ = 99% (*P*_h_ < 0.0001)], and −0.06 g/L [95% CI: −0.25, 0.14; *P* = 0.56; *I*^2^ = 64% (*P*_h_ = 0.04)], respectively.

**Figure 3:**
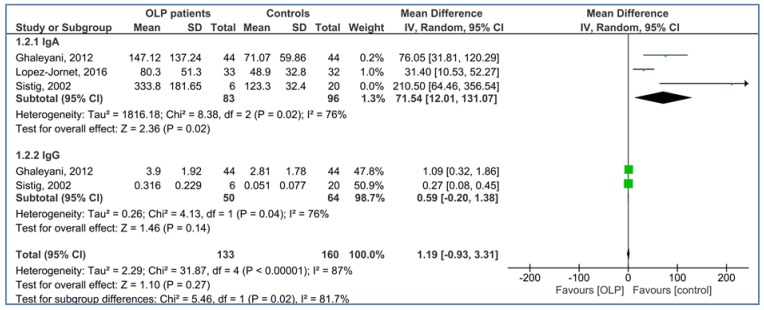
Forest plot of random-effects of salivary immunoglobulins (IgA and IgG) levels in oral lichen planus (OLP) patients compared to the healthy controls.

The comparison of salivary levels of IgA and IgG in the OLP patients as compared to the healthy controls is shown in Figure 3. The pooled MDs of salivary IgA and IgG levels were 71.54 mg/L [95% CI: 12.01, 131.07; *P* = 0.02; *I*^2^ = 76% (*P*_h_ = 0.02)] and 0.59 mg/L [95% CI: −0.20, 1.38; *P* = 0.14; *I*^2^ = 76% (*P*_h_ = 0.04)], respectively.

## 9. Quality Assessment

Based on NOS questionnaire for case-control studies, a mean score of 6.75 was achieved for the studies (Table 2).

## 10. Publication Bias

**Figure 4:**
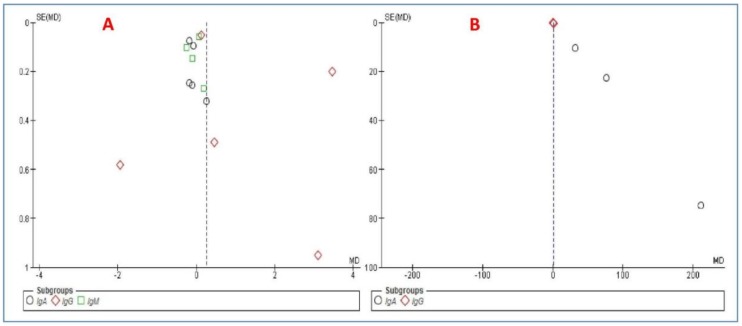
Funnel plot of (**A**) the serum levels and (**B**) salivary levels of immunoglobulins.

Figure 4 shows the funnel plot of the results of each analysis. There was no the publication bias based on the Begg’s and Egger’s tests (*P* > 0.05) between the studies about the results of the serum levels of Igs (Figure 4A) and salivary IgA (Figure 4B). These tests could not be run for the result of the salivary level of IgG because there were less than three studies in this subgroup analysis (Figure 4B).

## 11. Discussion

Two meta-analyses [22,23] showed that serum and salivary cytokines (interleukin-6 and tumor necrosis factor-alpha), as important immunological factors involved in systemic inflammation, were significantly more detected in the OLP patients as compared to the controls. The present meta-analysis checked the serum and salivary Igs levels in OLP patients compared to controls, and showed no elevation in the serum levels. However, there was an elevation in the salivary levels of IgA and IgG, unless only IgA values reached significant difference. Out of five studies reporting the serum IgA and IgG levels [15,16,17,19,20], only one [17] reported a significant difference for IgA level (a lowered level), while three studies [15,16,19] reported a significant difference for IgG level (an elevated level in two studies and a lowered level in the other) in OLP patients as compared to the healthy controls. Three studies [10,14,18] reporting salivary IgA level and two studies [14,18] reporting salivary IgG level reported an elevated level of both Igs and a significant difference in the OLP patients as compared to the healthy controls. One study [18] checked salivary subclasses of IgG and IgA in the OLP patients compared to healthy controls (a higher level in OLP for each subclass) and concluded that these salivary Ig could have a significant function in pathogenesis of OLP. The IgG and IgA levels obtained in the saliva samples were determined only in very few samples and could not be detected precisely in most of the samples, possibly because of low sensitivity of the equipment in measuring the Ig [20]. Other studies [10,14,18] used radial immunodiffusion test or enzyme-linked immunosorbent assay (ELISA) to detect salivary IgA and IgG, which showed a high detection in all samples. One study [24] reported the salivary IgA detection by ELISA method that had much smaller coefficients of variation than the single radial immunodiffusion method.

It is well-known that Igs concentrations both in serum and in saliva are extremely variable and, worthy of note, Igs levels can vary in healthy subjects in response to different stimuli such as rest versus physical exercise and oral probiotic intake, as well as simply on the basis of gender [9,25,26,27].

Racial, geographical, and environmental effects were found to impact the etiology of OLP. In addition, sampling, number of samples, endemic infections, dietary habits, and interfering factors such as age, sex, and clinical variants of OLP were other effective factors which were different among the studies. All factors could have caused the observed differences among studies, but further investigations are required to find the most important factors involved in the observed difference [15]. In this meta-analysis, it was found that the measurement method could be another factor to be considered.

Limitations: (i) there were a few studies on saliva; (ii) there were different measurement methods of Ig; (iii) heterogeneity was among most subgroups. Strengths: (i) there were the age- and sex-matching controls with the OLP patients in most of the studies; (ii) most of the studies had high quality.

## 12. Conclusions

Unless only a few studies were conducted on saliva, the results indicated that the Ig salivary levels had higher values than serum levels, and this was remarkable for IgA. Despite believing that future studies are required to evaluate salivary Ig levels, it seems that they have a significant function in pathogenesis of OLP. These studies should be aware of the effective factors involved in OLP in order to find better and more accurate results.

## Figures and Tables

**Table 1:** Characteristics of the studies included in the meta-analysis (*n* = 8).

First Author (year)	Country	Number of Patients/Mean Age (year)/Male%	Number of Controls/Mean Age (year)/Male%	IgA	IgG	IgM	Method
Scully, 1982 [16]	Scotland	35 42 years 48.6%	27 40 years 48.1%	Serum	Serum	Serum	Modified single radial immunodiffusion [21]
Sklavounou, 1983 [17]	Greece	50 53 years 40%	20 46.4 years 50%	Serum	Serum	Serum	The single radial immunodiffusion [21]
Lundström, 1985 [19]	Sweden	34 55.9 years 20.6%	23 56.4 years 26.1%	Serum	Serum	Serum	IgG (immunochemical turbidimetric) and IgA and IgM (electroimmuno Assay)
Sistig, 2002 [18]	Croatia	65 4 years 33.3%	20 37 years 50%	Saliva	Saliva	-	IgG (ELISA) and IgA (radial immunodiffusion)
Ghaleyani, 2012 [14]	Iran	44 45.6 years 34.1%	44 44.8 years 38.6%	Saliva	Saliva	-	IgA (Human IgA Saliva Diametra kit, the binding Site, Italy), and IgG (radial immunodiffusion)
Mehdipour, 2013 [15]	Iran	50 40 years 48%	25 37 years 48%	Serum	Serum	Serum	Autoanalyzer (Abbot- alcion, USA) and Pars Azmon kits
Divya, 2014 [20]	India	30 matched matched	30 matched matched	Serum	Serum	-	Dade Behring BN ProSpec Nephelometer (Sri Ramachandra University).
Lopez-Jornet, 2016 [10]	Spain	33 57 years 21.2%	32 53 years 25%	Saliva	-	-	ELISA kit (Bethyl, Montgomery, TX, USA)

Abbreviations: Ig, immunoglobulin; ELISA, enzyme-linked immunosorbent assay.

**Table 2:** Quality ratings for the studies included on the basis of Newcastle–Ottawa quality assessment scale (*n* = 8).

First Author (Year)	Selection				Comparability *		Outcome			Total Score	Quality
	Case Definition Adequate	Representativeness of the Cases	Selection of Controls	Definition of Controls	Main Factor	Additional Factor	Ascertainment of Exposure	Same Method of Ascertainment for Cases and Controls	Non-Response Rate		
Scully, 1982 [16]	*	*	-	*	*	*	*	*	-	7	Good
Sklavounou, 1983 [17]	*	*	-	*	*	-	*	*	-	6	Fair
Lundström, 1985 [19]	*	*	-	*	*	*	*	*	-	7	Good
Sistig, 2002 [18]	*	*	-	*	-	-	*	*	-	5	Fair
Ghaleyani, 2012 [14]	*	*	-	*	*	*	*	*	-	7	Good
Mehdipour, 2013 [15]	*	*	-	*	*	*	*	*	-	7	Good
Divya, 2014 [20]	*	*	*	*	*	*	*	*	-	8	Good
Lopez-Jornet, 2016 [10]	*	*	-	*	*	*	*	*	-	7	Good
**Mean Score**										6.75	

* One star for age-matching (Main Factor) and another star (Additional Factor) for sex-matching.
